# Intraoperative Endoluminal Pyloromyotomy Versus Stretching of the Pylorus for the Reduction of Delayed Gastric Emptying After Pylorus-Preserving Partial Pancreatoduodenectomy: A Blinded Randomized Controlled Trial (PORRIDGE Study; DRKS00013503)

**DOI:** 10.1245/s10434-025-16950-5

**Published:** 2025-02-04

**Authors:** Matthias C. Schrempf, Matthias Anthuber, Johann Spatz, Florian Sommer, Dmytro Vlasenko, Bernd Geissler, Sebastian Wolf, Stefan Schiele, David R. M. Pinto, Michael Hoffmann

**Affiliations:** 1 Department of General, Visceral and Transplantation Surgery, University Hospital Augsburg, Augsburg, Germany; 2 Department of General and Visceral Surgery, Barmherzige Brueder Krankenhaus Munich, Munich, Germany; 3 Department of General, Visceral and Thoracic Surgery, Asklepios Stadtklinik Bad Tölz, Bad Tölz, Germany; 4 Department of Computational Statistics and Data Analysis, Institute of Mathematics, University of Augsburg, Augsburg, Germany

**Keywords:** Delayed gastric emptying, Partial pancreatoduodenectomy, Postoperative complications, Pyloromyotomy, Pancreatic surgery, Randomized controlled trial

## Abstract

**Background:**

Pylorus-preserving partial pancreatoduodenectomy (ppPD) is a treatment for tumors of the pancreatic head. Delayed gastric emptying (DGE) is one of the most common complications following ppPD. In a retrospective analysis, intraoperative endoluminal pyloromyotomy (PM) was shown to be associated with a reduction in DGE rates.

**Objective:**

The aim of this randomized controlled trial was to investigate the effect of intraoperative endoluminal PM on DGE after ppPD.

**Methods:**

Patients undergoing ppPD were randomized intraoperatively to receive either PM or atraumatic stretching of the pylorus prior to creation of the duodenojejunostomy. The primary endpoint was the rate of DGE within 30 days after surgery.

**Results:**

Sixty-four patients were randomly assigned to the PM group and 64 patients were assigned to the control group. There were no differences between the two groups regarding baseline characteristics. The DGE rate was 59.4% (76/126). In two patients (1.6%) DGE was not assessable. The most common DGE grade was A (51/126, 40.5%), followed by B (20/126, 15.9%) and C (5/126, 4.0%). The rate of DGE was 62.5% in the PM group versus 56.3% in the control group (odds ratio 1.41, 95% confidence interval 0.69–2.90; *p* = 0.34). The complication rate did not differ between both groups (*p* = 0.79) and there were no differences in quality of life on postoperative day 30.

**Conclusions:**

Intraoperative endoluminal PM did not reduce the rate or severity of DGE after ppPD compared with atraumatic stretching of the pylorus.

**Supplementary Information:**

The online version contains supplementary material available at 10.1245/s10434-025-16950-5.

The standard treatment for resectable pancreatic cancer and other malignant tumors originating in the periampullary region is partial pancreatoduodenectomy (PD). Improvements in perioperative management and surgical technique have dramatically reduced mortality in recent decades, with in-hospital mortality rates in high-volume centers now at 1–5%.^1,2^ Despite this success, major pancreatic resections are still associated with significant morbidity.^3^ One of the most common complications after both PD and pylorus-preserving PD (ppPD) is delayed gastric emptying (DGE), with incidence rates of up to 61%.^4,5^ First described by Warshaw and Torchiana in 1985, there were several different definitions of DGE in the literature, which affected the comparability of clinical studies until 2007, when the International Study Group of Pancreatic Surgery (ISGPS) proposed a standardized definition and grading system.^6,7^ When DGE is present after pancreatic surgery, it can prevent patients from returning to a normal solid diet in a timely manner, affect quality of life, prolong the hospital stay, and increase treatment costs.^7,8^ Although the exact etiology remains unclear, it has been postulated that pyloric spasm, partial devascularization of the pylorus, and perioperative hormonal changes play a role in the development of DGE.^9–11^ Numerous risk factors for the occurrence of DGE have been identified, including patient age and body mass index (BMI), pre-existing conditions such as diabetes or the presence of a biliary stent, and postoperative intra-abdominal complications such as anastomotic leakage, pancreatic fistula, hematoma or abscess.^12–15^ Various modifications of surgical technique, including pyloric resection, route of reconstruction, and pyloric dilation, have been evaluated in several studies for their effects on DGE, with inconsistent results.^4,16,17^ Larger randomized controlled trials and high-quality meta-analyses using the ISGPS definition of DGE have failed to demonstrate an effect of surgical modifications.^14,15,18,19^

We introduced intraoperative endoluminal pyloromyotomy (PM) at our institution to reduce DGE and demonstrated an association with a lower rate of DGE in a retrospective study.^20^ These results encouraged us to further investigate our findings in a prospective randomized controlled trial (PORRIDGE trial, DRKS00013503).^21^ The aim of this study was therefore to investigate the impact of intraoperative endoluminal PM during ppPD on DGE compared with atraumatic stretching of the pylorus.

## Methods

The PORRIDGE study was a randomized, controlled, patient- and assessor-blinded study (RCT) with two parallel groups. The superiority hypothesis of this trial was that intraoperative endoluminal PM during ppPD is associated with a reduced DGE rate compared with multidimensional atraumatic stretching of the pylorus.

The study was conducted as a single-center study at the Department of General, Visceral and Transplant Surgery, University Hospital Augsburg, Augsburg, Germany.

Approval of the study protocol was granted by the Ethics Committee of the Ludwig Maximilians University Munich (reference number 17-605). The study was prospectively registered in a World Health Organization (WHO) primary registry on 27 December 2017 (German Clinical Trials Register, registration number DRKS00013503). The full WHO trial registration dataset is available via the WHO International Clinical Trials Registry Platform (ICTRP) search portal (https://trialsearch.who.int/). All participants were required to provide written informed consent prior to enrollment in the study. The Introduction and Methods sections are based on the published protocol that was published with open access under a Creative Commons license.^21^

### Patients and Eligibility Criteria

Patients were eligible for participation if they were 18 years of age or older and scheduled for elective ppPD, regardless of the underlying condition. Patients who were unable to give informed consent, patients under legal guardianship, or patients participating in other invention studies that could potentially affect the endpoint of this study were excluded from participation. Informed consent was obtained by surgeons at the University Hospital Augsburg.

### Randomization and Blinding

Participants were randomized intraoperatively after the absence of macroscopically visible metastases and technical resectability with pylorus preservation had been confirmed. Patients were randomized to either PM or pyloric stretching (for details see ‘surgical procedures’ below). In cases of early randomization, where neither study procedure could be performed, or if the decision for total pancreatectomy was made after randomization, the randomization was discarded and the participant was excluded from the study without further data collection according to the study protocol. Allocation to one of the two study arms was performed using a validated web-based randomization tool that generates a random allocation sequence (https://www.randomizer.at, provided by the Institute for Medical Informatics, Statistics and Documentation [IMI]) of the Medical University of Graz, Graz, Austria). An allocation ratio of 1:1 was applied using block randomization with a fixed block size. The block size was not disclosed throughout the study.

Patients and study personnel involved in data collection and endpoint assessment were blinded to allocation. Due to the nature of the study procedure, blinding of the operating surgeons was not possible.

### Surgical Procedures

**Fig. 1:**
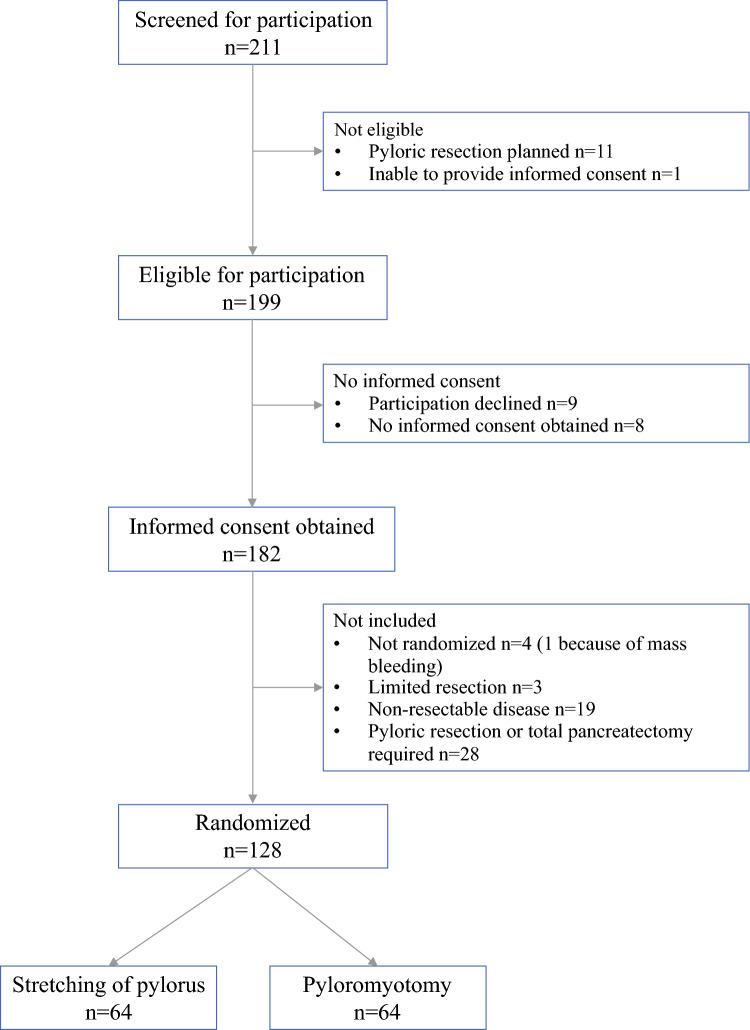
Trial flowchart

During ppPD, the duodenum was transected 2–4 cm distal to the pylorus using a linear stapler. Pancreaticojejunostomy and hepaticojejunostomy were created as end-to-side anastomoses. Depending on the results of the randomization, one of two different surgical maneuvers was performed prior to the creation of the duodenojejunostomy. Patients randomized to the PM group underwent endoluminal PM with electrocautery to transect the mucosa, submucosa, and circular pyloric muscle anteriorly and posteriorly at the 12 and 6 o’clock positions (electronic supplementary material [ESM] Fig. 1) as described previously.^20^ In patients assigned to the control group, the pyloric muscle was atraumatically stretched using Gross–Maier forceps before the duodenojejunostomy was created. The duodenojejunostomy was created as an end-to-side anastomosis with a single-layer monofilament atraumatic running suture. Reconstruction was performed in all patients using an omega loop in an antecolic fashion and a side-to-side Braun jejunostomy. A nasogastric tube (NGT) was placed in the stomach during the operation and was removed on the first postoperative day (POD) unless medical reasons prevented its removal. All patients received a subcutaneous 100 μg dose of octreotide intraoperatively, followed by a subcutaneous 100 μg dose of octreotide three times daily thereafter until POD 5, based on the internal standard.

### Primary Endpoint

The primary endpoint was the rate of DGE according to the ISGPS definition.^7^ The two study groups (pyloromyotomy vs. stretching of the pylorus) were compared with regard to the primary endpoint.

If the NGT was still in place or was re-inserted between PODs 4 and 7, or if the patient was unable to eat solid oral food until POD 7, DGE (grade A) was diagnosed. DGE was categorized as grade B if the NGT was still present or was re-inserted between PODs 8 and 14, or if the patient was unable to eat solid oral food until POD 14. In cases where the NGT was still present or was re-inserted after day 14, or the patient was unable to tolerate solid food until day 21, DGE was classified as grade C in accordance with the ISGPS definition.

### Secondary Endpoints

Secondary endpoints included DGE grade according to the ISGPS definition, operative time, estimated blood loss, complication rate, type of complications, and overall morbidity according to the Clavien–Dindo classification,^22^ in-hospital mortality, length of hospital stay, rate of primary DGE (defined as DGE in the absence of intra-abdominal complications), and postoperative quality of life. The European Organization for Research and Treatment of Cancer (EORTC) Quality of Life Questionnaire–Core 30 (QLQ-C30), a valid and widely used questionnaire in cancer patients,^23^ was used in combination with the supplementary module for pancreatic cancer patients (QLQ-PAN26) to assess quality of life. Quality-of-life data were collected preoperatively and postoperatively on days 7, 14, 30, and 90.

### Sample Size Calculation

Based on published data from our institution, we predicted a DGE rate of 40.9% in the PM group and 66.7% in the non-PM group. Based on these results, we calculated a sample size of 58 patients per group to ensure a power of 80% at a two-sided significance level of 5%. To compensate for possible dropouts, an additional 10% of patients were included in each group. Thus, 64 patients per group were enrolled, resulting in a total of 128 patients.

### Statistical Analysis

Depending on the distribution, continuous data are presented as mean ± standard deviation (SD) or median with interquartile range, and categorical data are presented as numbers with percentages. Approximately normally distributed continuous variables were compared using the independent *t* test, while non-normally distributed continuous variables were analyzed using the Mann–Whitney *U* test. Categorical data, including the primary endpoint, were compared using the Chi-square test. Fisher’s exact test was used for categorical data if the requirements for the Chi-square test were not met. A two-sided *p*-value < 0.05 was considered significant. Analysis of the primary endpoint was conducted as an intention-to-treat (‘as randomized’) analysis. Binary logistic regression analysis of the primary endpoint was performed, which included risk factors with a potential association with DGE (*p* < 0.15). Quality-of-life data were analyzed in accordance with the EORTC QLQ-C30 scoring manual.^31^ A subgroup analysis was performed for patients without intra-abdominal complications, to assess the rate of primary DGE in this patient population.

## Results

Between February 2018 and December 2023, 211 patients scheduled for partial PD were screened for participation. Of the screened population, 199 patients were eligible for participation, informed consent was obtained from 182 patients, and a total of 128 patients were intraoperatively randomized and assigned to one of the two study groups. Details are shown in the Consolidated Standards of Reporting Trials (CONSORT) flowchart (Fig. 1).

There were no differences in baseline demographic, clinical, and histologic characteristics between the PM and control groups (Table 1).

**Table 1:** Demographic and clinical characteristics

Characteristic	PM [*n* = 64]	Control [*n* = 64]	*p* value
Sex			
Female	24 (37.5)	31 (48.4)	0.21
Male	40 (62.5)	33 (51.6)	
Age, years	69.4 ± 8.9	67.4 ± 9.2	0.21
BMI ≥ 30 kg/m^2^	10 (15.6)	17(26.6)	0.13
Chronic renal insufficiency	3 (4.7)	4 (6.3)	1.0
Diabetes mellitus	10 (15.6)	10 (15.6)	1.0
Weight loss (≥10% within 6 months)	15 (23.4)	22 (34.4)	0.17
Neoadjuvant chemotherapy	3 (4.7)	3 (4.7)	1.0
ASA III or higher	37 (57.8)	35 (54.7)	0.72
PV/SMV involvement	5 (7.8)	1 (1.6)	0.21
Histology			0.33
Pancreatic ductal adenocarcinoma	30 (46.9)	34 (53.1)	
Distal bile duct cancer	9 (14.1)	12 (18.8)	
Ampullary cancer	7 (10.9)	8 (12.5)	
Duodenal cancer	1 (1.6)	0	
IPMN	6 (9.4)	1 (1.6)	
Chronic pancreatitis	2 (3.1)	1 (1.6)	
Other	9 (14.1)	8 (12.5)	

Data are expressed as mean ± SD or *n* (%)

*PM* pyloromyotomy, *BMI* body mass index, *ASA* American Society of Anesthesiologists, *PV* portal vein, *SMV* superior mesenteric vein, *IPMN* intraductal papillary mucinous neoplasm, *SD* standard deviation

### Outcomes

In two patients (1.6%), DGE was not assessable; one patient died before return to a solid diet was completed without meeting the criteria for DGE at the time of death, and one patient developed a lymphatic fistula that required temporary cessation of oral intake. Among the remaining 126 patients, the overall DGE rate was 60.3% (76/126). The rate of DGE was 62.5% in the PM group and 56.3% in the control group (odds ratio [OR] 1.41, 95% confidence interval [CI] 0.69–2.90; *p* = 0.34). DGE grade A was the most common DGE grade in the study population (51/126, 40.5%), followed by grade B (20/126, 15.9%) and grade C (5/126, 4.0%). There were no differences in the distribution of DGE grades (*p* = 0.89) or severe DGE (PM group 20.3% vs. control group 18.8%; *p* = 0.76) between both groups. A subgroup analysis was performed to assess the incidence of DGE in the absence of an intra-abdominal complication. In the subgroup of patients without intra-abdominal complications (*n* = 73), there were no differences in the frequency of primary DGE between the two groups (*p* = 0.29).

The median operating time was slightly longer in the PM group compared with the control group (351 min vs. 330 min; *p* = 0.0496) but there were no differences in the median estimated blood loss between both groups (600 mL vs. 500 mL; *p* = 0.5). The intraoperatively inserted NGT was removed after the same amount of time in both groups (*p* = 0.80). Re-insertion rates of the NGT (*p* = 0.84), time to solid food intake (*p* = 0.21), and length of postoperative stay (*p* = 0.82) were similar between the PM and control groups. In each group, six patients (9.4%) required a surgical revision. The results are shown in Table 2.

**Table 2:** Outcomes

	PM [*n* = 64]	Control [*n* = 64]	*p* value
DGE	40 (62.5)	36 (56.3)	0.34
Not assessable	2 (3.1)		
DGE grade^a^			0.89
A	27 (67.5)	24 (66.7)	
B	9 (22.5)	11 (30.6)	
C	4 (10)	1 (2.8)	
DGE^a^ grade B and C only	13 (20.3)	12 (18.8)	0.76
Primary DGE^b^	22/36 (61.1)	18/37 (48.6)	0.29
Operating time, min	351 (299–410)	330 (276–384)	0.0496
Estimated blood loss, mL	600 (400–875)	500 (400–800)	0.5
Removal of the first NGT, days	2.3 ± 5.0	2.1 ± 2.8	0.80
Re-insertion of the NGT	18 (28.1)	17 (26.6)	0.84
Solid food intake, days	10.4 ± 7.0	9.0 ± 4.1	0.21
Reoperation)	6 (9.4)	6 (9.4)	1.0
Postoperative stay, days	15 (13–20)	16 (12–23)	0.82

Data are expressed as mean ± SD, *n* (%), or median (IQR)

*PM* pyloromyotomy, *DGE* delayed gastric emptying, *NGT* nasogastric tube, *IQR* interquartile range, *SD* standard deviation

^a^Only patients with DGE were included in the analysis

^b^DGE in patients without intra-abdominal complications

Postoperative mortality was 3.1% (*n* = 4). A Clavien–Dindo complication grade of ≥III occurred in 39.8% of patients (*n* = 51). The overall Comprehensive Complication Index (CCI^®^) for the study population was 35.0 (SD 20.7). Intra-abdominal complications occurred in 54 patients (42.2%). The most common intra-abdominal complication was a postoperative pancreatic fistula (POPF; *n* = 24, 18.8%). The rate of POPF was the same in both groups (18.8% vs. 18.8%; *p* = 1.0). Of the 15 patients with postoperative bleeding, 3 patients had to be treated surgically, 3 patients underwent embolization, and 9 patients were treated endoscopically. There were no differences in mortality rate, frequency or type of complications, and the CCI^®^ between both groups (Table 3).

**Table 3:** Postoperative complications

Characteristic	PM [*n* = 64]	Control [*n* = 64]	*p*-Value
In-hospital mortality	3 (4.7)	1 (1.6)	0.62
30-day mortality	3 (4.7)	1 (1.6)	0.62
Clavien–Dindo ≥III complications	24 (42.2)	27 (37.5)	0.59
CCI^®^	36.4 (±21.8)	33.6 (±19.6)	0.79
Intra-abdominal complications	27 (42.2)	27 (42.2)	1.0
Biochemical leak	3 (4.7)	4 (6.3)	1.0
POPF	12 (18.8)	12 (18.8)	1.0
Grade B	7 (10.9)	11 (17.2)	0.31
Grade C	5 (7.8)	1 (1.6)	0.21
Intra-abdominal fluid collection	6 (9.4)	7 (10.9)	0.77
Bile leak	8 (12.5)	7 (10.9)	0.78
Chyle leak	5 (7.8)	5 (7.8)	1.0
Postoperative hemorrhage	6 (9.4)	9 (14.1)	0.41
Pulmonary aspiration and pneumonia	3 (4.7)	2 (3.1)	1.0
Pulmonary embolism	0 (0)	1 (1.6)	1.0
Surgical site infection	9 (14.1)	3 (4.7)	0.07
Urinary tract infection	1 (1.6)	5 (7.8)	0.21
DVT	0 (0)	1 (1.6)	1.0
NSTEMI	1 (1.6)	0 (0)	1.0
Portal vein thrombosis	1 (1.6)	0 (0)	1.0

Data are expressed as *n* (%) or mean (±SD)

*PM* pyloromyotomy, *CCI*^*®*^ Comprehensive Complication Index^®^, *POPF* postoperative pancreatic fistula, *DVT* deep vein thrombosis, *NSTEMI* non-ST elevation myocardial infarction, *SD* standard deviation

### Risk Factors for Delayed Gastric Emptying and Multivariable Analysis

Demographic factors and postoperative complications with a potential association (*p* < 0.15 in univariable analysis) with the primary endpoint as well as the study intervention itself were included in a multivariable analysis. The presence of a POPF was associated with an increased risk of DGE in the univariable analysis (OR 3.83, 95% CI 1.22–12.06; *p* = 0.016). In the logistic regression analysis for DGE, which included bile leaks, postoperative hemorrhage, reoperation, POPF, and the study intervention, the association between POPF and DGE failed to reach statistical significance (OR 2.79, 95% CI 0.79–9.82; *p* = 0.11). PM was not associated with a difference in DGE rate in the multivariable analysis (OR 1.48, 95% CI 0.70–3.12; *p* = 0.31). The results are shown in Table 4.

**Table 4:** Risk factors and protective factors for DGE

Variable	DGE [*n* = 76, 60.3%]	No DGE [*n* = 50, 39.7%]	Univariable *p* value	Multivariable OR (95% CI)	Multivariable *p* value
Age	69.1 ± 8.9	67.3 ± 9.5	0.30	–	
Male sex	43 (56.6)	28 (56.0)	0.95	–	
BMI ≥ 30 kg/m^2^	15 (19.7)	11 (22.0)	0.76	–	
ASA III or higher	43 (56.6)	27 (54.0)	0.78	–	
Diabetes mellitus	14 (18.4)	6 (12.0)	0.34	–	
Chronic renal insufficiency	6 (7.9)	1 (2.0)	0.24	–	
Operating time	354 ± 96	342 ± 71	0.44	–	
Previous abdominal surgery	22 (28.9)	20 (40.0)	0.20	–	
Malignant disease	59 (77.6)	41 (82.0)	0.55	–	
Chyle leak	7 (9.2)	2 (4.0)	0.32	–	
Intra-abdominal fluid collection	7 (9.2)	5 (10.0)	1.0	–	
Bile leak	12 (15.8)	3 (6.0)	0.10	2.15 (0.48–9.71)	0.32
POPF grade B or C	19 (25.0)	4 (8.0)	0.016	2.79 (0.79–9.82)	0.11
Postoperative hemorrhage	12 (15.8)	3 (6.0)	0.10	2.29 (0.57–9.29)	0.25
Reoperation	10 (13.2)	2 (4.0)	0.12	1.24 (0.19–8.22)	0.82
Pyloromyotomy performed	40 (52.6)	22 (44.0)	0.34	1.48 (0.70–3.12)	0.31

Data are expressed as mean ± SD or *n* (%)

*DGE* delayed gastric emptying, *OR* odds ratio, *CI* confidence interval, *BMI* body mass index, *ASA* American Society of Anesthesiologists, *POPF* postoperative pancreatic fistula

### Quality of Life

Preoperatively, the scores for the global health status did not differ between the two groups (PM 58.5 vs. control 61.6, range 0–100; *p* = 0.63). The preoperative physical functioning score was slightly higher in the PM group compared with the control group (85.5 vs. 76.9; *p* = 0.038). All other functional and symptom scores of the EORTC QLQ-C30 and PAN26 quality-of-life questionnaires showed no differences between the two groups preoperatively. Quality-of-life data for POD 30 are shown in ESM Table 1. There were no differences in the global health status scores between the two groups on POD 30. There was a trend towards a lower symptom burden for nausea and vomiting on POD 30 in the PM group, but the difference did not reach statistical significance (PM 11.6 vs. control 18.4, range 0–100; *p* = 0.051); this trend was absent on POD 90 (*p* = 0.54). All other functional and symptom scores did not show any differences between the two groups at POD 30.

## Discussion

This is the first RCT to investigate the effects of intraoperative endoluminal PM on DGE. In the PORRIDGE study, no significant difference in the DGE rate was found between patients who underwent endoluminal PM during ppPD and those who underwent atraumatic pyloric stretching. We found no difference in most secondary endpoints. The severity of DGE was also unaffected by PM.

The overall DGE rate in our study was 59.4%, which is slightly higher than the DGE rate we originally predicted but is in the upper range of published DGE rates for patients undergoing PD.^4^ One possible explanation is that the most common grade of DGE in our study was grade A, which may have been underestimated in the retrospective data used as the basis for the sample size calculation. The incidence of DGE grades B and C combined was 19.5%. No complications were attributable to the PM itself. In general, patients were given a clear liquid diet immediately after removal of the NGT tube. If the patients tolerated the clear liquid diet without nausea or vomiting, they were gradually switched from one day to the next to a liquid diet such as soup, then to a soft diet, and if they tolerated the soft diet, to a solid diet.

Sources of bias were minimized by intraoperative randomization and blinding. Since DGE was defined according to the ISGPS criteria, the assessment of DGE was based on clinical findings without the use of scintigraphy, which is considered by some authors to be the gold standard for the diagnosis of gastroparesis.^24,25^ The study was conducted at a single center, which may limit the generalizability of the results due to internal standards that may not apply to other institutions. We routinely administered somatostatin analogs for 5 PODs after PD. Regarding the potential effects of somatostatin analogs on DGE, a meta-analysis of 12 clinical studies including 11 randomized controlled trials and 1 non-randomized trial, found a significant reduction in pancreatic fistulas and postoperative hospital stay after pancreatic surgery, with no effect on the incidence of DGE.^26^

The results of the PORRIDGE study contrast with a retrospective study in 110 patients that our group published 2021, which showed a significant reduction in DGE rates after intraoperative endoluminal PM.^20^

The concept of PM is becoming increasingly established in patients with gastroparesis.^27^ G-POEM (gastric peroral endoscopic myotomy) has been validated as successful treatment for this patient population in numerous retrospective and one prospective randomized controlled trials.^27–29^ In surgical patients, the concept was first described by Kim,^30^ who performed a Fredet–Ramstedt PM in combination with an arthroplasty in a series of 47 consecutive ppPD patients. Although a different definition of DGE was used, the authors showed a reduction of DGE compared with patients without PM from the same institution.

There could be several reasons why no effect of PM on DGE was found in this study. The pathophysiology of DGE is largely unknown. The common hypothesis is that functional impairment of gastric motility and pyloric function plays a major role in DGE.^4,15^ Hormonal factors, local ischemia, neuronal damage to the antrum and pylorus as well as resection of the duodenal pacemaker have been proposed as potential causes.^4^

Intra-abdominal complications have been identified as a main risk factor for DGE after PD.^14^ Some authors suggest the term ‘secondary’ DGE to describe this situation.^31^ However, DGE is a common problem even in the absence of surgical complications, hence there must be other factors contributing to the development of DGE. In our study, about half of the patients with DGE had an intra-abdominal complication, while the other half had ‘primary’ DGE.

Four randomized trials have compared the effects of pylorus resection and pylorus preservation on DGE.^15,24,32,33^ Only one of these studies found a difference in DGE rates, while the PROPP trial by Hackert et al., the largest RCT on this topic,^15^ as well as the most recent RCT by Busquets et al.,^24^ found no association between pyloric resection and DGE. The results of these RCTs in combination with our findings indicate that pyloric spasm or a mechanical problem involving the pylorus may not be the main contributor to the development of DGE and suggest a more complex etiology of DGE.

## Conclusion

In this randomized controlled trial, PM during ppPD did not reduce the incidence or severity of DGE compared with atraumatic stretching of the pylorus. There was no difference in quality of life in the early postoperative period.

## Supplementary Information

Supplementary file 1

Supplementary file 2

## Data Availability

A fully anonymized dataset as well as the statistical code can be made available upon justified scientific request and after ethical approval has been granted. Depending on the extent of the data use and the planned research, appropriate credit or co-authorship must be granted to the authors of this study. Any requests should be addressed to the corresponding author.
