# Supplementary figures and images for: Intraoperative Endoluminal Pyloromyotomy Versus Stretching of the Pylorus for the Reduction of Delayed Gastric Emptying After Pylorus-Preserving Partial Pancreatoduodenectomy: A Blinded Randomized Controlled Trial (PORRIDGE Study; DRKS00013503)
Source: Ann Surg Oncol. 2025 Feb 4;32(6):4076–84. doi: 10.1245/s10434-025-16950-5 (PMC12049319; doi:10.1245/s10434-025-16950-5)

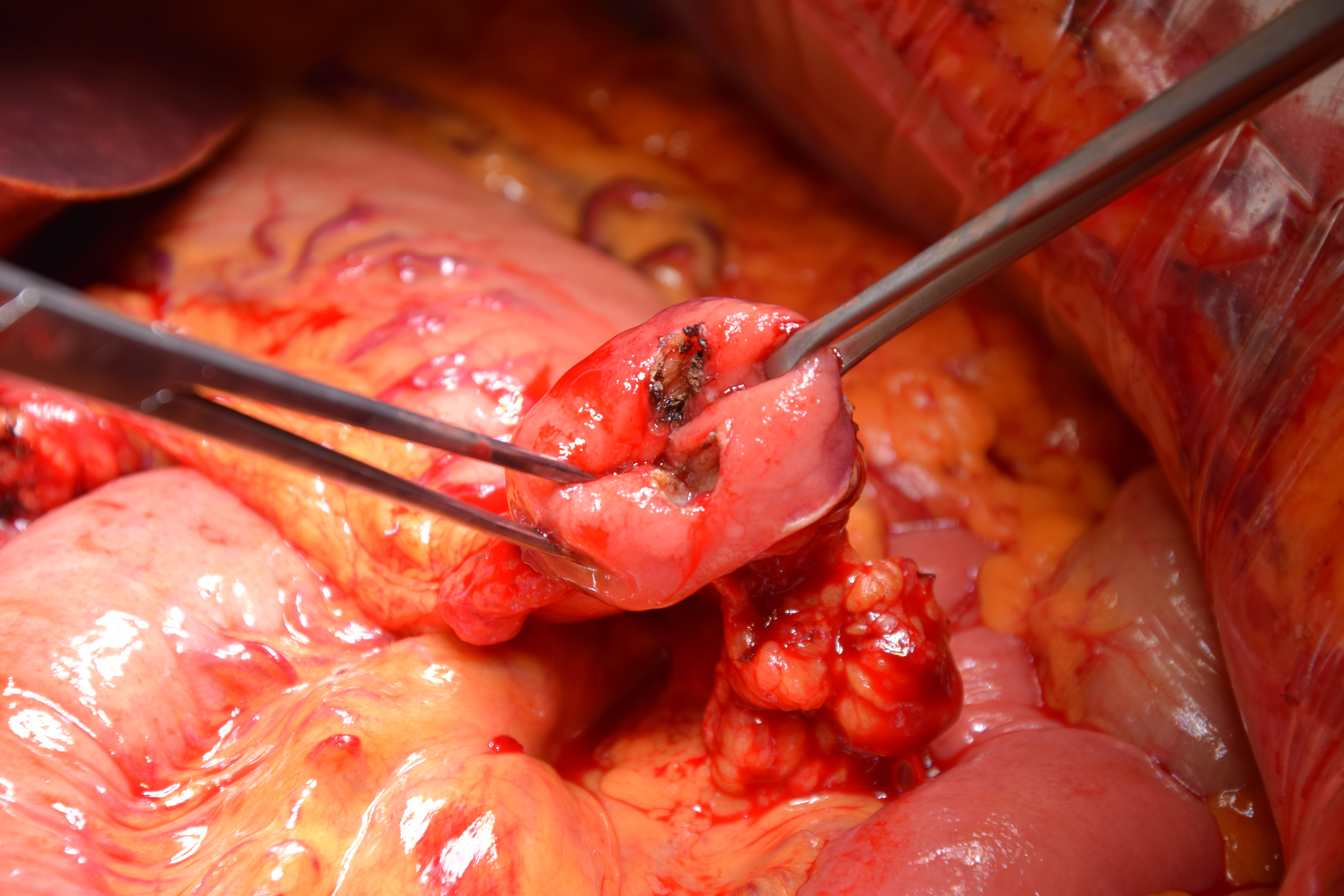

Supplement: Supplementary file 1 [file 10434_2025_16950_MOESM1_ESM.jpg]
