# Supplementary material for: Intraoperative Endoluminal Pyloromyotomy Versus Stretching of the Pylorus for the Reduction of Delayed Gastric Emptying After Pylorus-Preserving Partial Pancreatoduodenectomy: A Blinded Randomized Controlled Trial (PORRIDGE Study; DRKS00013503)
Source: Ann Surg Oncol. 2025 Feb 4;32(6):4076–84. doi: 10.1245/s10434-025-16950-5 (PMC12049319; doi:10.1245/s10434-025-16950-5)
Supplement: Supplementary file 2 [file 10434_2025_16950_MOESM2_ESM.docx]

Supplemental Table 1 EORTC QLQ-C30 and PAN26 Quality of Life on postoperative day 30

| QLQ-C30 item / PAN26 item | Control  n=47 | PM  n=45 | Univariable  *P* | Direction |
| --- | --- | --- | --- | --- |
| Global health status / QoL | 47.8 (19.9) | 44.8 (18.4) | 0.50 | ↑ |
| Physical functioning | 49.2 (24.3) | 51.6 (21.7) | 0.56 | ↑ |
| Role functioning | 34.8 (29.5) | 32.9 (35.4) | 0.51 | ↑ |
| Emotional functioning | 54.4 (26.9) | 53.4 (29.1) | 0.91 | ↑ |
| Cognitive functioning | 70.6 (22.6) | 72.6 (29.1) | 0.40 | ↑ |
| Social functioning | 46.0 (31.5) | 50.4 (38.9) | 0.62 | ↑ |
| Fatigue | 62.0 (26.3) | 63.5 (27.8) | 0.80 | ↓ |
| Nausea / Vomiting | 18.4 (21.5) | 11.6 (19.8) | 0.051 | ↓ |
| Pain | 44.2 (30.3) | 43.2 (31.4) | 0.86 | ↓ |
| Dyspnea | 38.3 (32.6) | 31.9 (35.5) | 0.27 | ↓ |
| Insomnia | 46.8 (33.8) | 44.4 (37.6) | 0.72 | ↓ |
| Appetite loss | 50.7 (34.2) | 56.3 (34.0) | 0.46 | ↓ |
| Constipation | 24.8 (32.2) | 16.7 (30.1) | 0.12 | ↓ |
| Diarrhea | 16.3 (26.8) | 9.6 (23.2) | 0.13 | ↓ |
| Financial difficulties | 23.5 (28.4) | 17.0 (29.8) | 0.12 | ↓ |
| Pancreatic pain | 43.4 (24.5) | 42.4 (23.4) | 0.81 | ↓ |
| Bloating | 37.7 (29.5) | 49.2 (35.6) | 0.13 | ↓ |
| Digestive Symptoms | 50.0 (31.7) | 60.1 (32.4) | 0.14 | ↓ |
| Taste | 44.4 (34.8) | 45.9 (34.3) | 0.85 | ↓ |
| Indigestion | 37.8 (33.8) | 33.3 (36.2) | 0.45 | ↓ |
| Flatulence | 39.9 (31.9) | 42.4 (34.0) | 0.73 | ↓ |
| Weight loss | 42.8 (38.3) | 55.6 (38.3) | 0.11 | ↓ |
| Weakness | 51.4 (32.7) | 54.5 (35.3) | 0.63 | ↓ |
| Dry mouth | 46.7 (32.1) | 53.3 (37.2) | 0.38 | ↓ |
| Hepatic symptoms | 19.9 (26.2) | 14.3 (24.3) | 0.22 | ↓ |
| Altered bowel habits | 35.1 (29.4) | 24.8 (26.8) | 0.08 | ↓ |
| Body image | 46.2 (31.3) | 43.2 (29.1) | 0.74 | ↓ |
| Side-effects | 55.3 (29.6) | 55.0 (34.0) | 0.98 | ↓ |
| Future Worries | 64.4 (32.9) | 72.6 (27.8) | 0.27 | ↓ |
| Planning of activities | 61.6 (32.9) | 59.1 (37.3) | 0.80 | ↓ |
| Satisfaction with health care | 67.8 (30.4) | 77.8 (25.7) | 0.11 | ↑ |
| Sexuality | 63.4 (35.6) | 44.4 (40.4) | 0.07 | ↑ |
| Date are mean (SD), range for each score is 0 - 100  PM pyloromyotomy group, QLQ-C30 Quality of Life questionnaire Core 30, PAN26 pancreatic module  ↑ higher number indicates better function / better QoL  ↓ lower number indicates less symptoms / better QoL | | | | |
